# Record linkage without patient identifiers: Proof of concept using data from South Africa’s national HIV program

**DOI:** 10.1371/journal.pgph.0004835

**Published:** 2025-07-09

**Authors:** Khumbo Shumba, Jacob Bor, Cornelius Nattey, Dickman Gareta, Evelyn Lauren, William Macleod, Matthew P. Fox, Adrian Puren, Koleka Mlisana, Dorina Onoya

**Affiliations:** 1 Health Economics and Epidemiology Research Office, Faculty of Health Sciences, University of the Witwatersrand, Johannesburg, South Africa; 2 Department of Global Health, Boston University School of Public Health, Boston, Massachusetts, United States of America; 3 Department of Epidemiology, Boston University School of Public Health, Boston, Massachusetts, United States of America; 4 Africa Health Research Institute (AHRI), Research Data Department, Durban, South Africa; 5 Institute of Social and Preventive Medicine, University of Bern, Bern, Switzerland; 6 Graduate School for Health Sciences, University of Bern, Bern, Switzerland; 7 School of Laboratory Medicine and Medical Sciences, University of KwaZulu Natal, Durban, South Africa; 8 Department of Biostatistics, Boston University School of Public Health, Boston, Massachusetts, United States of America; 9 National Health Laboratory Service, Centre for HIV and STIs, National Institute for Communicable Diseases, Johannesburg, South Africa; 10 School of Laboratory Medicine and Medical Sciences, University of KwaZulu Natal, Durban, Johannesburg, South Africa; University of California San Francisco, UNITED STATES OF AMERICA

## Abstract

Linkage between health databases typically requires patient identifiers such as names and personal identification numbers. We developed and validated a record linkage strategy to combine administrative health databases without identifiers for South Africa’s public sector HIV program. We linked CD4 counts and HIV viral loads from South Africa’s TIER.Net with the National Health Laboratory Service (NHLS) database for patients receiving care between 2015–2019 in Ekurhuleni District (Gauteng Province). Linkage variables were result value, specimen collection date, facility of collection, year and month of birth, and sex. We used three matching strategies: exact matching on exact values of all variables, caliper matching allowing a ± 5 day window on result date, and specimen barcode matching using unique specimen identifiers. A sequential linkage approach applied specimen barcode, followed by exact, and then caliper matching. Exact and caliper matching were validated using barcodes (available for 34% of records in TIER.Net) as a “gold standard”. Performance measures were sensitivity, positive predictive value (PPV), share of patients linked, and percent increase in data points. We attempted to link 2,017,290 laboratory test results from TIER.Net (523,558 unique patients) with 2,414,059 NHLS test results. Exact matching achieved 69.0% sensitivity and 95.1% PPV. Caliper matching achieved 75% sensitivity and 94.5% PPV. Sequential linkage matched 41.9% using specimen barcodes, 51.3% through exact matching, and 6.8% through caliper matching, for 71.9% (95% CI: 71.9, 72.0) of test results matched overall, with 96.8% (95% CI: 96.7, 97.1) PPV and 85.9% (95% CI: 85.7, 85.9) sensitivity. This linked 86.0% (95% CI: 85.9, 86.1) of TIER.Net patients to the NHLS (N = 1,450,087), increasing laboratory results in TIER.Net by 62.6%. Linkage of TIER.Net and NHLS without patient identifiers attained high accuracy and yield without compromising privacy. The integrated cohort provides a more complete laboratory test history and supports more accurate HIV program indicator estimates.

**Key message**

- Clinical data linkage without identifiers is a useful tool for researchers and policymakers to analyze health-related data while protecting patient information.
- We linked data from South Africa’s TIER.Net clinical database and the National Health Laboratory Service (NHLS) National HIV Cohort without patient identifiers, protecting patient confidentiality.
- A sequential approach – initially matching on specimen barcode (where available), and in their absence, using laboratory result value, specimen collection date, facility of collection, patient year and month of birth, and sex – linked 86% of TIER.Net patients with the NHLS data with a 3.2% type I error rate (1 – PPV).
- Integrating TIER.Net with the NHLS National HIV Cohort increased the number of HIV monitoring laboratory results by 62.6% relative to TIER.Net alone.
- Piloted in Ekurhuleni, a large urban district, this linkage approach offers novel opportunities to evaluate South Africa’s public sector HIV program using an integrated NHLS-TIER.Net HIV cohort.

## Background

South Africa’s HIV treatment program is the largest in the world, with about 5.2 million adult patients on antiretroviral therapy (ART) in 2019 [1]. Although mass provision of ART has reduced HIV-related morbidity and mortality [2–6], HIV remains the fifth leading cause of death in South Africa (4.8% of deaths in 2019) [7], ranked fourth in Africa (5.6% of deaths in 2019) [8], with over 200,000 new infections annually in the country [9].

South Africa’s public sector HIV program is monitored via two administrative databases: the “Three interlinked electronic registers” (TIER.Net) clinical database [10–14] and the National Health Laboratory Service (NHLS) National HIV Cohort [15–24]. The NHLS Cohort is the primary laboratory database including all data generated by public-sector medical laboratories while TIER.Net contains data generated by clinical events at all HIV management centres in South Africa. Unlike TIER.Net, NHLS data is nationally de-duplicated, enabling longitudinal analyses even when patients re-enter care at other facilities [16,21]. Both national in scope, TIER.Net and NHLS contain complementary data that clinicians draw on for patient care. However, the two databases are not currently integrated, and no consistent patient identifier exists to enable patient-level longitudinal analyses using information from both data sources.

Linkage of health databases traditionally requires primary personal identifiers such as names, national identification numbers, addresses, and phone numbers. However, with heightened concern for data privacy in South Africa and elsewhere, access to patient-identifying information is increasingly restricted to clinical management purposes [25,26]. Therefore, validated techniques are required to link databases without primary patient identifiers [27–29] to enable program monitoring, evaluation, and research. A variety of privacy-preserving record linkage (PPRL) approaches have been proposed, with most involving the encryption of primary identifiers behind data owners’ firewalls and linkage of those encoded data [30]. Here, we explore the feasibility of linkage without primary patient identifiers at all, relying instead on laboratory event information recorded in both databases. Our paper builds on prior efforts by Edward Nicol et al. [31] and Ingrid Bassett et al. [32] to link the NHLS with HIV patient management systems for specific clinical cohorts in South Africa, which were limited by small cohort sizes, the lack of scalable linkage methods that do not rely on patient identifiers, and limited validation of linkage strategies.

In this paper, we set out to develop and validate a linkage strategy to link TIER.Net and the NHLS Cohort without patient identifiers. As a proof of concept, we used data from Ekurhuleni district, a large, mostly urban district in Gauteng province, South Africa. We developed multiple linkage approaches, validated their performance against “gold standard” data, and quantified the benefits of linkage with respect to the completeness of the resulting database. This study differs from prior work by implementing a scalable, privacy-preserving approach for integrating large administrative health databases at scale, a method not previously applied in South Africa’s public HIV program. Our goal was to create an integrated HIV cohort with comprehensive clinical and laboratory data that would enable longitudinal analyses of the full HIV care cascade not possible with NHLS or TIER.Net data alone. This integrated cohort offers opportunities to improve HIV care and monitoring at a national scale while prioritizing both accuracy and the protection of patient privacy.

## Methods

This paper details the methodology and approach used for record linkage in accordance with recommendations for reporting of linkage methodology [33,34] and reporting guidelines for observational research [35].

### Study design, setting and study population

This was record linkage study using routine secondary laboratory test (CD4 count and viral load) data from South Africa’s HIV program. The study population was all patients receiving HIV care in Ekurhuleni District from 1 January 2015 – 31 December 2019 at 102 public-sector health facilities with at least one CD4 count or HIV viral load, to link and identify all their laboratory tests in the NHLS. Ekurhuleni District is situated adjacent to the City of Johannesburg, the City of Tshwane, and the Sedibeng District Municipality. The district’s HIV prevalence rose from 12% in 2012 to 14% in 2017 [36]. The district is served by a network of healthcare facilities, including a district hospital, community health centres, and primary health clinics.

### Data sources

In this study, we compiled all available CD4 and viral load data on this study population from two sources: TIER.Net and NHLS, which we accessed for research purposes on 01 October 2020.

### Three Interlinked Electronic Registers (TIER.Net)

TIER.Net is South Africa’s facility-based electronic patient health data management system. Established in 2010 and scaled up in the following years, TIER.Net serves as the primary monitoring platform for the national HIV care and treatment program [10]. Data from patient charts are captured into TIER.Net by clinic staff. TIER.Net contains data on clinical events including ART initiation, ART pick-up dates, regimen type, and clinic visits (Table 1). While laboratory tests (CD4 counts and HIV viral loads) information is also captured, the process is manual and inconsistent, resulting in incomplete laboratory test results in TIER.Net [14]. TIER.Net is not nationally networked [10], and test results preceding HIV diagnosis and ART initiation are largely unavailable on TIER.Net [37,38]. The TIER.Net patient ID is allocated by facilities, and patients who seek care at alternative facilities receive a new TIER.Net patient ID, creating duplicate records that hinders patient tracking across facilities [14].

**Table 1:** Comparison of data availability in TIER.Net and NHLS databases.

Data element	TIER.Net	NHLS
Month and year of birth	√	√
Sex	√	√
Facility name	√	√
HIV test results	√	–
Baseline WHO HIV clinical staging	√	–
ART Start date	√	–
Result date	√	–
Taken date	–	√
Test value	√	√
ART regimen type	√	–
ART pickup date	√	–
Clinic appointment dates	√	–

Notes: “√” indicates the availability and “-” unavailability of the specific data element in the respective database. Result date (TIER.Net) and taken date (NHLS) are synonymous.

### National Health Laboratory Service (NHLS) National HIV Cohort

The NHLS provides all laboratory and pathology services for the country’s public sector HIV care and treatment program [39]. The NHLS maintains a centralised database of all laboratory test data (including CD4 count and HIV viral load (VL) data), with results logged to the NHLS Corporate Data Warehouse (CDW). While the NHLS processes and houses all the laboratory test data, it lacks other clinical details, which are captured in TIER.Net (Table 1). The NHLS’s CDW previously developed a de-duplication record linkage algorithm for its laboratory test records [15]. More recently, a team at NHLS, University of Witwatersrand, and Boston University developed, implemented, and validated an improved record-linkage algorithm with much higher sensitivity, enabling analysis of the NHLS database as a national cohort covering all lab-monitored patients in South Africa’s public sector HIV program [15,16]. The NHLS National HIV Cohort has been used to track trends in CD4 counts at presentation, assess retention in care regardless of patient transfer, quantify treatment outcomes for different groups, and evaluate the impact of HIV policy changes [16–23].

### Linkage variables

#### Demographic and laboratory test variables.

De-identified data were extracted from the TIER.Net and NHLS databases by the Ekurhuleni department of health and NHLS CDW data team. Extracted data were laboratory event-specific demographic data [year of birth (YOB), month of birth (MOB), and sex of the patient], facility data (province name, district name, sub-district name, and health facility name), and details on all CD4 counts and HIV viral loads (result value, test date, and test type) taken between January 1, 2015 and December 31, 2019. The details of each of these linking variables are provided in Box 1. All variables were harmonized between the databases to ensure equivalent formatting for linkage. We linked health facilities starting with a crosswalk provided by the National Institute for Communicable Diseases (NICD) at NHLS, which ensured that any changes in facility names were updated and standardized. We then manually reviewed facility names within Ekurhuleni District to ensure correspondence to the existing Department of Health’s list. This ensured that all health facility names were standardized to be the same across the two databases. We also retained de-identified unique patient IDs from each database. From TIER.Net, we extracted the “TIER.Net ID”. From NHLS, we extracted a unique patient ID created by NHLS CDW (henceforth, “NHLS CDW ID”) as well as the National HIV Cohort ID (henceforth, “NHLS Cohort ID”). At the time of writing, the NHLS Cohort ID was available only through March 2018.

#### Specimen barcodes as a gold standard matching variable.

Some TIER.Net laboratory results were recorded with their NHLS specimen barcode. These alphanumeric barcodes are allocated centrally by NHLS and are not duplicated within or across health facilities. Barcodes are affixed to biological specimens (e.g., blood test tubes), the corresponding NHLS test request form, the facility’s specimen register, and provided with the test results sent back to facilities from NHLS. The barcode is the same across all the tests performed on the same biological specimen. Except for cases where the same test was repeated on the same specimen, the combination of barcode and test type is unique. Barcodes are available for nearly all laboratory results in the NHLS database. Although the barcode data are highly incomplete in TIER.Net and cannot be used as the only linkage strategy, the combination of barcode and test type offers a highly accurate “gold standard” for validation of other approaches.

To confirm the suitability of using barcodes as a gold standard, we assessed the concordance of other test information (YOB, MOB, sex, test date and value, and facility name) when barcodes matched. We first excluded test results where the specimen barcodes and test type were not unique (0.013% -TIER.Net and 0.005% - NHLS). We then identified test results with the same barcode and test type in the two databases and quantified the % discordance in the associated test information. To assess the probability of barcode matching by chance, we randomly selected 100,000 test results from TIER.Net and linked them to 100,000 randomly sampled test results from NHLS (S1 Table). The expectation was that a very small share of these randomly selected pairs would be true matches. We quantified the proportion of discordance in the randomly-matched pairs. By comparing the share of barcode matches that were fully discordant in the other characteristics with the share that would be expected to be fully discordant due to random chance, we were able to estimate the false positivity rate in the barcode matching, under the assumption that all fully discordant records were different individuals. This is an upper bound on the false positivity rate, given that some of the fully discordant barcode matches actually may have been true matches with a lot of typographic error.

**Box 1. Description of the linkage variables.**

The following variables were used to link laboratory tests in TIER.Net and the NHLS National HIV Cohort:

- *Specimen Barcode –* Each blood specimen is assigned a unique NHLS specimen barcode.
- *Test type* – CD4 count or HIV viral load.
- *Test facility –* Facility where a specimen was taken (NHLS) or recorded (TIER.Net).
- *Test date –* “Taken date” in NHLS and “result date” in TIER.Net.
- *Test result value –* Numeric value of CD4 count or viral load test result. Some viral loads were classified as “lower than detectable limit”; these were coded as “0” for linkage to ensure that any variability in thresholds was addressed by standardizing these values across the datasets.
- *Year and month of birth –* Extracted from the date of birth recorded for each NHLS specimen and each TIER.Net patient record.
- *Biological Sex –* Recorded in TIER.Net and NHLS as “Male” or “Female”.

#### Exclusions before linkage.

**Fig 1:**
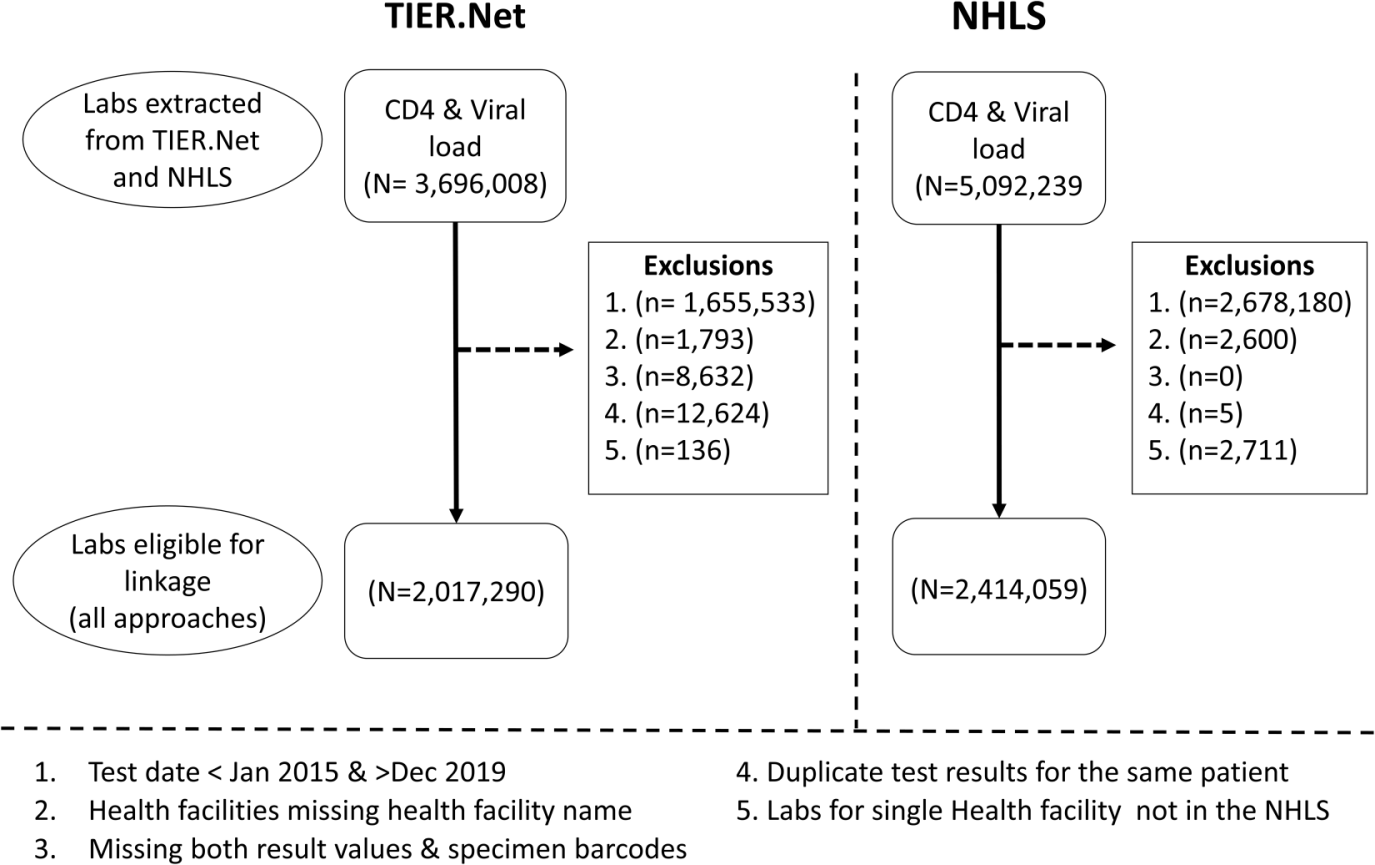
Flow diagram: laboratory results from TIER.Net and NHLS submitted for linkage.

At the time of analysis, we had access to TIER.Net and NHLS test results from 2004-2020. Our linkage was performed on all CD4 count and viral load tests in TIER.Net and NHLS with a test date between 1 January 2015 and 31 December 2019, subject to the following exclusions (**Fig 1**): (1) Health facilities not found in both databases; (2) test result entries with missing result values and duplicate test results per TIER.Net patient. The remaining laboratory results were considered “eligible test results” for inclusion in the analysis. Stata v16.1 was used in all data processing and analyses.

### Methods for record linkage

We applied four record linkage approaches using laboratory test result data.

(a)  ***Barcode matching:*** Matching based on the specimen barcode, and test type.
(b) ***Exact matching***. Matching on exact values of the linkage variables: YOB, MOB, facility name, sex, test date, test type, and test result value (with lower than detectable viral loads were coded as 0 in both datasets).
(c) ***Caliper matching.*** Matching using exact values of sex, facility name, test type, and test value while allowing for approximate matches on test date (±5 days). Viral load test results can be denoted as being “lower than detectable” (LDL) based on the sensitivity cut-offs of the testing methods, which may differ across laboratories and over time [40]. Since multiple patients may have VLs under LDL for tests requested on the same date and in the same facility, these test results may not be considered unique identifiable for linkage. We therefore coded all viral load “LDL” entries as “0” for linkage and validated the linking of these test results by testing the uniqueness of variable combinations (sex, test date, test facility, YOB and MOB) excluding the result value.
(d) ***Sequential linkage.*** Finally, we implemented the three methods above sequentially, using the most accurate method available for each record pair to maximize yield while maintaining high accuracy (matching by specimen barcodes where available, then the exact matching strategy and lastly caliper-matching for the remaining unmatched data).

### Evaluating performance of the linkage methods

We used the subset of laboratory tests with specimen barcodes in TIER.Net and NHLS to assess the performance of the exact and caliper matching linkage strategies. We assumed that barcodes were missing completely at random. Record pairs where the specimen barcode matched were defined as “true matches”. Record pairs where the specimen barcode did not match were defined as “true non-matches”. Performance was assessed across four dimensions: sensitivity, positive predictive value (PPV), linkage yield and enrichment of the TIER.Net laboratory profile because of the linkage. Using our calculations of sensitivity and PPV from the “gold standard” dataset, we then estimated these parameters for the complete eligible TIER.Net and NHLS test result data when linked using the four methods separately using conditional probabilities (S1 Text): barcodes matching, exact matching, caliper matching, and sequential linkage. We additionally assessed “yield” and “enrichment” (sensitivity analysis) for each method. Definitions are provided below:

(a) ***Sensitivity*.** We computed the sensitivity as the proportion of “true matches” (i.e., barcode matches) that were identified by each linkage approach.
(b) ***Positive predictive value (PPV).*** We computed PPV as the proportion of matches identified by each linkage approach that were “true matches”.

The approach for estimation of these parameters for the sequential linkage is provided in S1 Text. For sensitivity and PPV, we estimated exact binomial 95% confidence intervals using the one-sample Clopper–Pearson method [41]. Because there are a very large number of true non-matches, specificity and negative predictive value are nearly 100% and are not reported [21].

(c) ***Linkage yield*.** We defined linkage yield at both the laboratory test result and patient levels for tests conducted between 2015 and 2019. Test-level yield was defined as the proportion of TIER.Net laboratory results that could be linked to NHLS. We defined patient-level yield as the proportion of TIER.Net patients with laboratory results that were linked to NHLS based on at least one test result. We note that yield is distinct from sensitivity, which uses as a denominator the share of true matches between the datasets.
(d) ***Enrichment (Sensitivity analysis)*.** To assess the robustness of our linkage strategy in generating a more comprehensive dataset, we conducted a sensitivity analysis by enriching the TIER.Net database with additional laboratory results from the NHLS National HIV Cohort. The NHLS National HIV Cohort de-duplicated ID was available from January 2004 to March 2018. For each linkage strategy, we identified and extracted all laboratory results in the NHLS National HIV Cohort (Jan 2015 – Dec 2018) that were associated with that TIER.Net-linked NHLS Cohort ID. This linkage enriched the TIER.Net data with additional laboratory results not existing in TIER.Net, creating a more comprehensive profile of CD4 counts and HIV viral loads for patients in TIER.Net. We defined enrichment of the TIER.Net database as the percentage increase in the number of CD4 counts and viral loads associated with patients in TIER.Net after the linkage with the NHLS National HIV Cohort, relative to the number of CD4 counts and viral loads originally in TIER.Net alone.

Linkage yield and enrichment were calculated on the complete set of TIER.Net records with laboratory results included in the linkage exercise.

### Ethics approval and consent to participate

Ethical approval, including a waiver of informed consent to participate permitting the access to retrospective anonymized data, was obtained from the Medical Human Research Ethics Committee (Medical HREC) of the University of Witwatersrand, South Africa (HREC no. M200447) and the Boston University Medical Campus Institutional Review Board (IRB number: H-33442) in the United States. Permissions to access, analyse, and link de-identified patient-level TIER.Net data were obtained from the Gauteng provincial Department of Health (NHRD reference number: GP 202005–024) and the NHLS (AARMS permission number: PR2010539). As per South Africa’s POPI Act [21,25], only de-identified data were provided by the NHLS and the Ekurhuleni district for this study. All methods were performed in accordance with relevant ethical guidelines and regulations.

## Results

### Description of the data used for linkage

After applying the exclusion criteria on the 3,696,008 TIER.Net test results from the 2004–2020 data extract, a total of 2,017,290 “eligible TIER.Net test results” (668,900 CD4 counts and 1,348,390 viral loads) for the 523,558 patients in TIER.Net from 2015-2019 were available for linkage. Of these eligible TIER.Net test results, 605,506 (30.0%) were LDL viral loads. Fig 1 presents the data selection flow diagram. After the same exclusions were applied to 5,097,555 NHLS test results from 2004-2020, a total of 2,414,059 “Eligible NHLS test results” (836,633 CD4 counts and 1,577,426 viral loads) for the 985,734 non-unique NHLS patients from 2015-2019 were available for linkage. Of these NHLS test results, 688,277 (28.5%) were LDL viral loads. Overall, 684,973 (34.0%) of the eligible TIER.Net test results had specimen barcodes, compared to 2,410,230 (99.8%) of the eligible NHLS test results (Table 2). S1 Fig shows the number of test results in TIER.Net and NHLS in Ekurhuleni District over time. NHLS had more test results than TIER.Net throughout the study period, with about 25% more than TIER.Net in 2015 and about 15% more in 2019.

**Table 2:** Description of TIER.Net and NHLS datasets used in the linkage.

Parameter	TIER.Net	NHLS
*January 2015 – December 2019*		
Total number of test results #	2,017,290	2,414,059
Viral loads # (%)	1,348,390 (66.8%)	1,577,426 (65.3%)
CD4 counts # (%)	668,900 (33.2%)	836,633 (34.7%)
Test results with barcodes # (%)	684,973 (34.0%)	2,410,230 (99.8%)
Number of patients with test results # (ID)	523,558 (TIER_ID)	985,734 (CDW_ID)
*January 2015 – March 2018*		
Total number of test results #	1,257,913	1,545,467
Number of patients with test results # (ID)	403,476 (TIER_ID)	703,231 (CDW_ID) 479,883 (Cohort_ID)

Note: The table provides a summary of the NHLS and TIER.Net data used in the record linkage. Data are for patients receiving care at 102 facilities in Ekurhuleni province, January 2015 – December 2019. TIER_ID = unique patient identifier in TIER.Net; CDW_ID = unique patient identifier created by the NHLS Corporate Data Warehouse; Cohort_ ID = improved unique patient identifier created for the NHLS National HIV Cohort.

### Validation of the barcode as a suitable gold standard

Linking the TIER.Net and NHLS data using only the specimen barcode linked 608,210 (88.8%; 95% CI: 88.7-88.9) of TIER.Net test data that had a barcode (n = 684,973) (S1 Table**).** Among the test results with matching barcodes and test types, only 4.3% were discordant across more than one other characteristic (YOB, MOB, sex, test date and value, and facility name), and just 0.03% were discordant across all six characteristics. To validate the use of barcodes as a gold standard, we considered random matching as a critical control to evaluate their robustness and estimate the probability of false positives and discordant matches. We randomly matched 100,000 test results from TIER.Net to 100,000 test results from NHLS to assess the probability of discordant characteristics occurring by chance. Among these random pairs, 99.9% were discordant on two or more characteristics and 52.7% were discordant on at least four characteristics. Under the assumption that all fully discordant pairs were different individuals (even if there was a barcode match), we estimated that 0.06% (0.03/52.7) of all barcode matches were false positives. This assumption aimed to minimize the overestimation of linkage errors while maintaining robustness in the linkage methods applied. However, further investigation showed that the combination of barcode and test type achieved a PPV exceeding 99.9%, with false positives accounting for less than 0.01%. This provided a highly accurate variable pair for minimising potential mismatches and making it a suitable gold standard for reliable validation of the other approaches. However, the sensitivity was relatively low at 37% highlighting the importance of additional linkage strategies.

### Linkage approaches that uniquely identify laboratory results within each database

To identify potentially effective linking strategies, we assessed what share of test results were uniquely identified in TIER.Net. The combination of test type, test date, facility, sex, YOB and MOB uniquely identified a higher proportion of test results in both TIER.Net and NHLS (TIER.Net = 99.0%, NHLS = 99.1%) compared to replacing YOB and MOB with age at test (TIER.Net = 92.1%, NHLS = 91.6%). This difference was particularly important for the linkage of LDL viral loads, which lacked discriminating information in the result value field (Table 3). These investigations guided our selection of variables for exact and caliper matching.

**Table 3:** Percent of test results uniquely identified based on the following characteristics.

	Within TIER.Net		Within NHLS	
	All test results	LDL VL only	All test results	LDL VL only
Number of test results	2,017,290	605,506	2,414,059	688,277
*Of which, the number of test results (%)uniquely identified by:*				
(a) Test type, date, facility	435,778 (21.6%)	182,953 (30.2%)	245,790 (10.2%)	115,435 (16.8%)
(b) Test type, date, facility, sex, age	1,801,993 (89.3%)	557,568 (92.1%)	2,138,481 (88.6%)	630,540 (91.6%)
(c) Test type, date, facility, sex, YOB, MOB	1,989,169 (98.6%)	599,981 (99.1%)	2,376,874 (98.5%)	681,363 (99.0%)
(d) Test type, date, facility, sex, age, Result value	1,986,155 (98.5%)	577,466 (95.4%)	2,358,088 (97.7%)	632,642 (91.9%)
(e) Test type, date, facility, sex, YOB, MOB, result value	2,011,903 (99.7%)	602,435 (99.5%)	2,407,320 (99.7%)	681,607 (99.0%)

Note: YOB = Year of birth; MOB = Month of birth. LDL VL = Lower than Detectable Limit Viral Load: refers to viral load test results that were below the detection threshold of the laboratory assay. TIER.Net = Three Interlinked Electronic Registers: a clinical monitoring database used in South Africa’s public health sector for managing HIV care and treatment data. NHLS = National Health Laboratory Service: South Africa’s national laboratory network responsible for providing diagnostic and monitoring laboratory services, including HIV-related tests (CD4 counts and viral load). The table illustrates the different linkage variable combinations considered in our linkage strategies for the entire dataset and LDL viral loads.

### Sensitivity and positive predictive value for exact and caliper matching vis-à-vis barcode “gold standard” matching using NHLS-TIER.Net validation data

We evaluated exact and caliper matching using barcodes as a gold standard. We identified 608,210 test records with identical barcodes in TIER.Net and NHLS and considered these to be “true matches”. All other pairs of laboratory records from TIER.Net and NHLS in which barcodes differed (and were non-missing) were considered “true non-matches”. Exact matching yielded a total of 441,300 matches, of which 419,658 were “true matches”, a sensitivity of 69.0% (95% CI: 68.9, 69.1) and a PPV of 95.1% (95% CI: 95.0, 95.2). Caliper matching yielded a total of 487,077 matches of which 460,288 were “true matches”, with a sensitivity of 75.7% (95% CI: 75.6, 75.8) and a PPV of 94.5% (95% CI: 94.4, 94.6).

### Linkage performance and yield in the complete data

**Fig 2:**
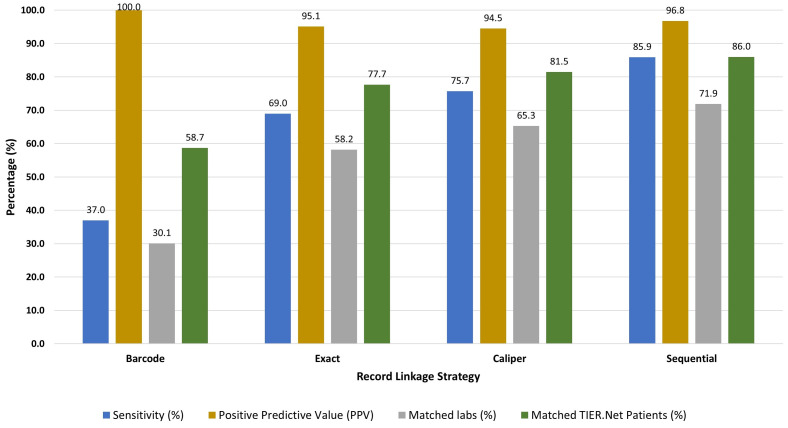
Record linkage performance for each linkage strategy in the full dataset. Note: The figure compares the estimated performance of each linkage strategy with respect to sensitivity, positive predictive value (PPV), lab-level linkage yield, and patient-level linkage yield. Estimates were based on extrapolation from the barcode subsample, under the assumption that barcodes were missing completely at random (S1 Text). Overall, the sequential linkage approach outperformed the other approaches with the highest linkage yield at the test (71.9%) and patient level (86.0%), high sensitivity (85.9%), and PPV (96.8%).

Of all eligible TIER.Net test results (N = 2,017,290), 608,210 (30.1%) were matched on barcodes, representing 25% of the eligible NHLS test results (N = 2,414,059) (Fig 2; S2 Table). Since all barcode matches were gold standard matches, PPV of this strategy was 100%, but sensitivity was estimated at just 37% (95% CI: 36.9, 37.1) and 58.7% (95% CI: 58.6, 58.8) of TIER.Net patients were matched. Exact matching linked 1,174,983 test results; 58.2% (95% CI: 58.2, 58.3)of TIER.Net test results and 77.7% (95% CI: 77.6,77.8) of TIER.Net patients were linked using this approach. Caliper matching had a greater yield with 1,317,429 matches, 65.3% (95% CI: 65.2, 65.4) of all TIER.Net test results and 81.5% (95% CI: 81.4, 81.6) of TIER.Net patients with test results captured between 2015 and 2019.

The sequential linkage approach combined all three strategies to maximize yield. Among the eligible test results, the sequential linkage matched 1,450,787 records, of which 608,210 were matched by barcode, 743,993 were exact matched, and 98,584 were caliper matched (and were not exact matches). This approach yielded the highest linkage yield, at 71.9% (95% CI: 71.9, 72.0) of TIER.Net test results matched. Overall, 86% (95% CI: 85.9, 86.1) of all TIER.Net patients with a CD4 count or viral load could be matched to the NHLS database using this approach. We estimated a sensitivity of 85.9% (95% CI: 85.8, 86.1). We estimated PPV at 96.8% (95% CI: 96.7, 96.9), (corresponding to a Type 1 Error Rate (1-PPV) of 3.2%), implying that just 3.2% of matches were false positives (Fig 2; S2 Table).

### Enrichment of TIER.Net through linkage to the NHLS National HIV Cohort

**Fig 3:**
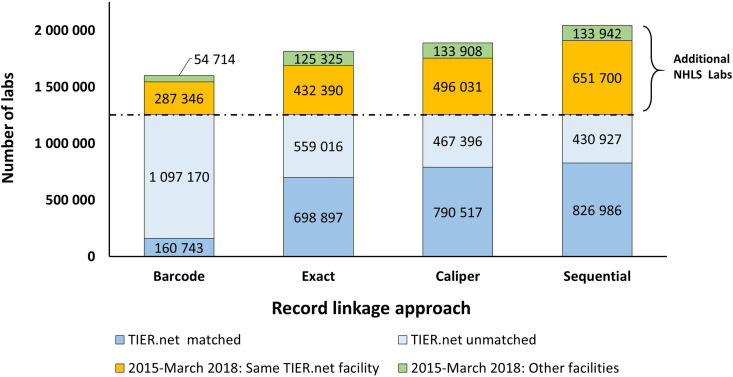
Enrichment of test results in TIER.Net through linkage with NHLS National HIV Cohort (test data generated between January 2015-March 2018).

At the time of writing, the NHLS National HIV Cohort ID was available for laboratory tests conducted between January 2004 and March 2018 (Table 1). The total number of additional test results identified for the period spanning January 2015-March 2018 from the NHLS National HIV Cohort for each strategy varied. Barcode matching led to a 27.2% increase (n = 342,060) in test results relative to TIER.Net alone. Exact matching led to a 44.6% increase (n = 560,715). Caliper matching led to a 50.1% increase (n = 629,939), while sequential linkage led to a 62.5% increase in the number of CD4 counts and viral loads relative to TIER.Net alone (n = 785,642). These numbers mean that before the linkage to the NHLS National HIV Cohort, TIER.Net contained only 61.6% of all available CD4 counts and viral loads conducted between January 2015-March 2018. Of the 785,642 CD4 counts, and viral loads newly identified through linkage to the NHLS National HIV Cohort, 133,942 (17.0%) were conducted at different facilities than the linking test event from the TIER.Net facility (Fig 3).

## Discussion

We developed and validated an algorithm to link two large administrative health databases - TIER.Net and the NHLS National HIV Cohort -without patient identifiers such as names or national ID numbers. The choice of record linkage methods depends on the objectives of the study [27,28]. Our goal was to leverage the comprehensive laboratory data available in the NHLS National HIV Cohort to complete the laboratory test profile for HIV patients observed in TIER.Net using privacy-preserving linkage methods. South African studies have linked the NHLS data with other patient registries before the enactment of the Protection of Personal Information Act South Africa (POPIA) [42–46]. However, some studies have attempted to link patient management systems in South Africa without patient identifiers, these efforts were constrained by small cohort sizes, the absence of scalable linkage methods, and limited validation of linkage strategies [31,32]. None have addressed these challenges by using large-scale data, making our study a significant step forward in developing and validating scalable linkage strategies without patient identifiers.

We established that a combination of individually non-identifying variables including basic demographic fields (year/month of birth and sex), health facility, and laboratory result (test type, test date, and test value) can be used to link the TIER.Net and NHLS databases with high accuracy and substantial linkage yield. A sequential record linkage strategy, leveraging barcodes for about a quarter of the data, linked 86% of TIER.Net patients to the NHLS database with a type 1 error rate of less than 4%. Combining TIER.Net with the NHLS National HIV Cohort increased the number of laboratory results of patients in TIER.Net by 62.5% relative to TIER.Net alone, indicating that the NHLS Cohort fills significant gaps in patient information in TIER.Net. Because our approach protects patient confidentiality and privacy, it may be generalizable in contexts where access to primary patient identifiers is limited. Our sensitivity analyses provided evidence of the robustness of our sequential linkage strategy. Enriching the TIER.Net database with data from the NHLS National HIV Cohort not observed in TIER.Net, demonstrated that the linkage strategy could generate a more comprehensive dataset, despite some limitations in sensitivity.

This study has several limitations. First, the use of barcodes as a gold standard for validation assumes that they are error-free, which may not always hold true due to occasional barcode mislabeling or data entry errors. Second, several facilities could not be linked between TIER.Net and NHLS and were excluded, highlighting the need for a national, regularly maintained crosswalk with NHLS and Department of Health facility identifiers. Third, the risk of false discovery-where records are incorrectly linked-remains despite a high PPV, as some discordant matches may reflect true linkages affected by data quality issues. Fourth, the NHLS National HIV Cohort was created using a validated algorithm, and like all de-duplication efforts contains some matching errors. Fifth, not all laboratory results in TIER.Net could be linked to NHLS. We were unable to accurately link 28% of CD4 count and viral load results in TIER.Net to NHLS. We cannot be sure why they were not linked. However, other studies have noted inconsistencies between information recorded in patient files and that captured in TIER.Net [13,14,38,47]. Sixth, the assumption that barcodes were missing completely at random was not tested due to data limitations. Future studies could use statistical tests to analyse the missingness patterns to validate this assumption. Seventh, our approach requires the availability of data on the same patient characteristics – here, laboratory test results – to facilitate linkage, and would not be suitable for linking databases that do not contain some shared data points. Eighth, the study was limited to one mostly urban district in South Africa, although the methods are likely generalizable more broadly. Lastly, potential biases need to be acknowledged, including selection bias (e.g., unlinked patients may differ systematically from linked patients), information bias (e.g., differences in how data were captured in TIER.Net and NHLS), and confounding factors that were not accounted for in our analysis. In light of these limitations, our linkage strategy may only be generalizable in the South African context due to the specific data sources we used.

The findings of this study contribute significantly to the understanding of privacy-preserving record linkage in resource-limited settings. In South Africa, where patient identifiers are not readily available for research purposes this study demonstrates the feasibility of linking large-scale clinical and laboratory data. Regionally, it provides a framework for other sub-Saharan African countries facing similar challenges. Globally, the study adds to the growing body of evidence supporting privacy-preserving linkage methods, showing that they can balance data integration and confidentiality without compromising accuracy

## Conclusion

Despite the exploratory nature of our study, the findings offer an exciting and readily available template for rapid integration of the NHLS National HIV Cohort and TIER.Net patient management system without compromising patient privacy and confidentiality for HIV research and policy evaluation in South Africa. Because 14% of TIER.Net patients with laboratory results – and all TIER.Net patients without laboratory results – remained unlinked, other methods, including the use of patient identifiers, should be used to create a comprehensive database for patient care and monitoring purposes.

### Patient and public involvement

The study design was informed by past interactions with patients and healthcare providers, but patients and/or the general public were not directly involved in the research’s design, implementation, or reporting.

## Supporting information

S1 Text Sensitivity and PPV estimation for “sequential linkage”. (DOCX)

S1 Fig CD4 and Viral load testing total volumes in Ekurhuleni District of Gauteng province, 2015-2019. Note: S1 Fig shows the number of laboratory test results in TIER.Net and NHLS datasets from 102 included facilities in Ekurhuleni District between January 2015 and December 2019. NHLS had more laboratory test volumes results than TIER.Net throughout the study period, with about 25% more than TIER.Net in 2015 and about 15% more in 2019. UTT = “universal test and treat” policy which eliminated CD4 criteria for ART eligibility; SDI = “same day initiation” policy under which patients were started on treatment on the date of clinical diagnosis.
(TIF)

S1 Table Difference across matching characteristics for TIER.Net-NHLS matched laboratory test records with specimen barcodes (randomly linked sample and all specimen barcode linked laboratory test records). (XLSX)

S2 Table Estimated performance of record linkage approaches in the full dataset. (XLSX)

## Abbreviations

AARMS: Academic Affairs and Research Management System
ART: antiretroviral therapy
CDW: Corporate Data Warehouse
CI: confidence interval
HIV: human immunodeficiency virus
HREC: Human Research Ethics Committee
ID: identification number
LDL: lower than detectable
MOB: month of birth
NHLS: The National Health Laboratory Service
NICD: National Institute for Communicable Diseases
POPIA: Protection of Personal Information Act
PPRL: privacy-preserving record linkage
PPV: positive predictive value
TIER.Net: Three interlinked Electronic Registers
YOB: year of birth.
