# Supplementary material for: Record linkage without patient identifiers: Proof of concept using data from South Africa’s national HIV program
Source: PLOS Glob Public Health. 2025 Jul 9;5(7):e0004835. doi: 10.1371/journal.pgph.0004835 (PMC12240394; doi:10.1371/journal.pgph.0004835)
Supplement: S1 Text — (DOCX) [file pgph.0004835.s001.docx]

**S1 Text. Sensitivity and PPV estimation for “sequential linkage”**

To estimate sensitivity and positive predictive value (PPV) for the whole dataset, we leveraged our estimates from the subsample of labs with barcodes, under the assumption that the presence (or absence) of barcodes was randomly assigned.

**Positive predictive value** is the probability that a match identified by a particular linkage approach is truly a match. Let “TrueMatch” reflect the actual match status (0=No, 1=Yes) for a pair of lab results, which may or may not be observed. Let “HasBarcodes” indicate whether “TrueMatch” is observed. The assumption that barcodes are missing completely at random (MCAR) is equivalent to the assumption that “HasBarcodes” is independent of true match or exact match status. Thus, under MCAR, we can write PPV of a given linkage strategy (e.g. exact match) as:

| $\text{PP}\text{V}_{\text{exact,HasBarcode}}\text{ =}\text{Pr} \left( \text{ }\text{TrueMatch}\text{=1} \right\vert\text{HasBarcode}\text{=1, }\text{ExactMatch}\text{=1)}$ |
| --- |
| $\text{ =}\text{Pr} \left( \text{ }\text{TrueMatch}\text{=1} \right\vert\text{HasBarcode}\text{=}0\text{, ExactMatch=1)}$ |
| $\text{ }\text{ }\text{ }\text{ =}\text{Pr} \left( \text{ }\text{TrueMatch}\text{=1} \right\vert\text{ExactMatch}\text{=1)}$ |
| $\text{ }\text{ =}\text{ PP}\text{V}_{\text{exact}}$ |

Similarly, for the caliper linking strategy, $\text{PP}\text{V}_{\text{caliper}}=\text{PP}\text{V}_{\text{caliper}\text{,HasBarcode}}$. For the barcode linking strategy, $\text{PP}\text{V}_{\text{barcode}}=100\%$ for record-pairs with barcodes. For pairs without barcodes, $\text{PP}\text{V}_{\text{barcode}}$ is undefined.

**Sensitivity** is the probability that a record pair that is a true match is also identified by a particular linkage approach. Again, we only observe the subset of true matches where both records have a barcode. Under the MCAR assumption, sensitivity of a given linkage strategy (e.g. caliper match) is the same where barcodes exist as where they don’t because the presence of barcodes is independent of match status, e.g.: $\text{Sen}_{\text{caliper,HasBarcode}\text{ }}=\text{Sen}_{\text{caliper,}\text{No}\text{Barcode}\text{ }}=\text{Sen}_{\text{caliper}}$ and similarly for $\text{Sen}_{\text{exact}}$.

The sensitivity of the barcode-linking strategy in the subsample with barcodes is $\text{Sen}_{\text{barcode}}\text{=} \Pr\left( \text{BarcodeMatch}\text{=1}\text{ } \right|\text{TrueMatch}\text{=1}\text{, }\text{HasBarcode}\text{=1}\text{)}=100\%$. However, in the full dataset, there are many true matches that are not part of the gold standard because they do not have barcodes. Thus, in the full dataset $\text{Sen}_{\text{barcode}}\text{=} \Pr\left( \text{BarcodeMatch}\text{=1} \right|\text{TrueMatch}\text{=1)}=\frac{\#BarcodeMatch}{\#TrueMatch}$. To estimate $\text{Sen}_{\text{barcode}}$, we first need to estimate #TrueMatch, which we can do based on our estimate of Sensitivity from the other linking strategies. $\text{Sen}_{\text{caliper}}=\frac{\#CaliperMatch AND TrueMatch}{\# TrueMatch}=\frac{\#CaliperMatch*PPV_{caliper}}{\# TrueMatch}$. Therefore, $\# TrueMatch=\frac{\#CaliperMatch*PPV_{caliper}}{Sen_{caliper}}$, and this estimate can then be used to obtain $\text{Sen}_{\text{barcode}}$ for the full dataset.

For the **sequential linkage approach**, we estimated PPV and Sen as the weighted averages of the component approaches. Because exact matching is nested within calliper matching and the two are not independent, we simplified the sums as the weighted averages of the barcode and caliper approaches.

The overall (PPV*_seq_*) of the sequential approach was estimated as:

$$\text{PP}\text{V}_{\text{seq}}\text{ }\text{= }\text{Pr}\text{(}\text{Match}_{\text{bar}}\text{|} \text{Match}_{\text{seq}}\text{)}\text{ }\text{×}\text{ }\text{PP}\text{V}_{\text{ba}\text{rcode,HasBarcodes}\text{r}}\text{ }$$

$$\text{+}\text{ }\text{Pr}\text{(}\text{Match}_{\text{cal}\text{iper}} \left| \text{ }\text{Match}_{\text{seq}} \right)\text{ × }\text{PP}\text{V}_{\text{cal}\text{iper,NoBarcode}}$$

where $\text{PP}\text{V}_{\text{ba}\text{rcode,HasBarcodes}\text{r}}\text{=1}$ and $\text{PP}\text{V}_{\text{cal}\text{iper,NoBarcode}}=\text{PP}\text{V}_{\text{cal}\text{iper,HasBarcodes}}$.

The overall (Sen*_seq_*) of the sequential approach was estimated as:

$$\text{Se}\text{n}_{\text{seq}}\text{ }\text{= }\text{Pr}\text{(}\text{Match}_{\text{bar}}\text{|} \text{Match}_{\text{seq}}\text{)}\text{ }\text{×}\text{ Se}\text{n}_{\text{ba}\text{rcode,HasBarcodes}\text{r}}\text{ }$$

$$\text{+}\text{ }\text{Pr}\text{(}\text{Match}_{\text{cal}\text{iper}} \left| \text{ }\text{Match}_{\text{seq}} \right)\text{ × }\text{Se}\text{n}_{\text{cal}\text{iper,NoBarcode}}$$

where $\text{Se}\text{n}_{\text{ba}\text{rcode,HasBarcodes}\text{r}}\text{=1}$ and $\text{Se}\text{n}_{\text{cal}\text{iper,NoBarcode}}=\text{Se}\text{n}_{\text{cal}\text{iper,HasBarcodes}}$.

***Source:*** *Molinaro AM. Diagnostic tests: how to estimate the positive predictive value. Neuro-Oncology Practice. 2015 Dec 1;2(4):162-6.*
