# Supplementary figures and images for: Record linkage without patient identifiers: Proof of concept using data from South Africa’s national HIV program
Source: PLOS Glob Public Health. 2025 Jul 9;5(7):e0004835. doi: 10.1371/journal.pgph.0004835 (PMC12240394; doi:10.1371/journal.pgph.0004835)

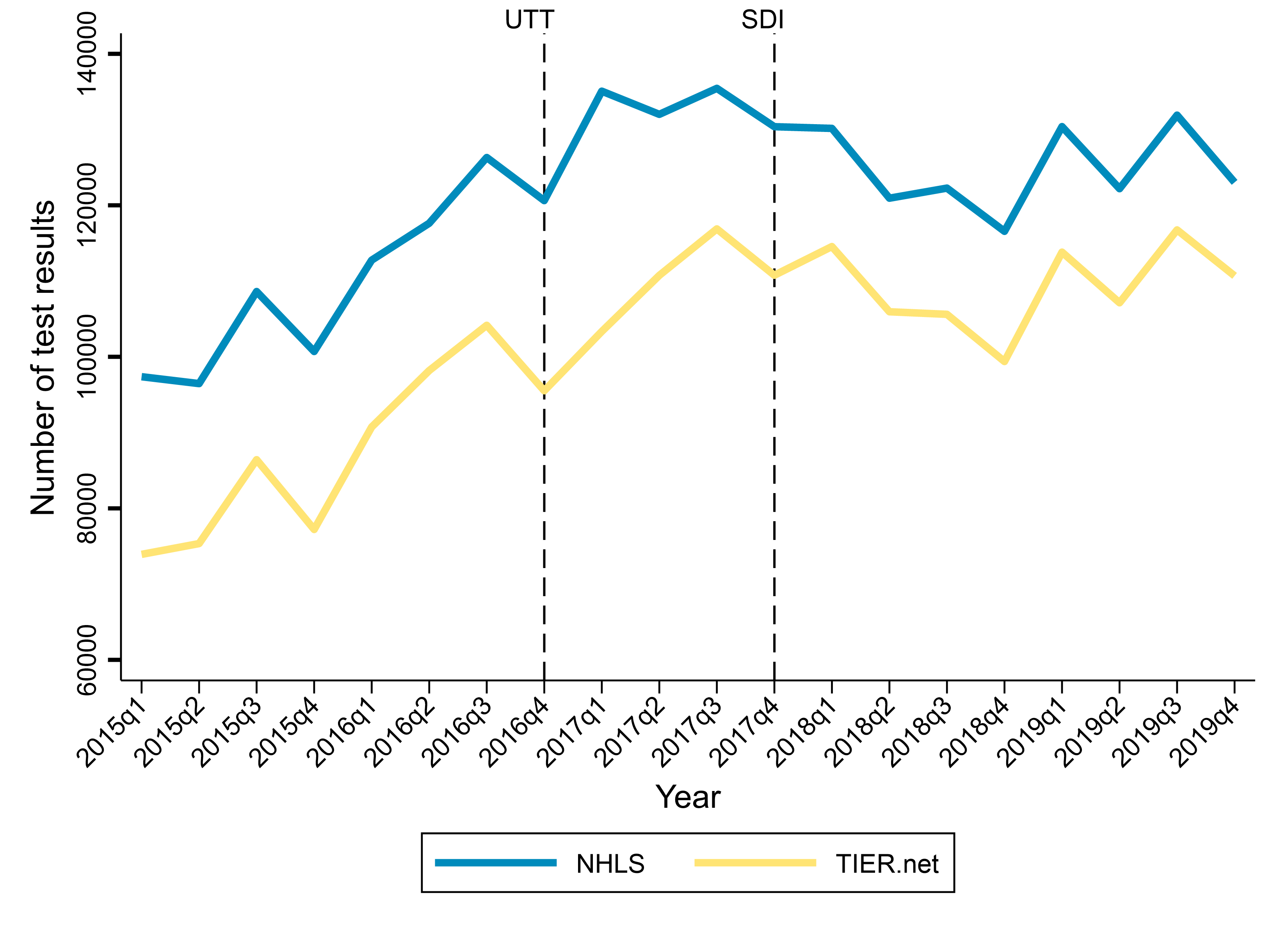

Supplement: S1 Fig — Note: S1 Fig shows the number of laboratory test results in TIER.Net and NHLS datasets from 102 included facilities in Ekurhuleni District between January 2015 and December 2019. NHLS had more laboratory test volumes results than TIER.Net throughout the study period, with about 25% more than TIER.Net in 2015 and about 15% more in 2019. UTT = “universal test and treat” policy which eliminated CD4 criteria for ART eligibility; SDI = “same day initiation” policy under which patients were started on treatment on the date of clinical diagnosis. (TIF) [file pgph.0004835.s002.tif]
